# Alcohol consumption and self-reported (SF12) physical and mental health among working-aged men in a typical Russian city: a cross-sectional study

**DOI:** 10.1111/add.12257

**Published:** 2013-07-05

**Authors:** Agnete S Dissing, Artyom Gil, Katherine Keenan, Jim McCambridge, Martin McKee, Alexey Oralov, Lyudmila Saburova, David A Leon

**Affiliations:** Department of Public Health, The University of CopenhagenCopenhagen, Denmark1; London School of Hygiene and Tropical MedicineLondon, UK2; Izhevsk State Technical UniversityIzhevsk, Russian Federation3

**Keywords:** Alcohol, mental health, physical health, quality of life, Russia, self-reported health, SF12

## Abstract

**Aim:**

To investigate the association between patterns of alcohol consumption and self-reported physical and mental health in a population with a high prevalence of hazardous drinking.

**Design:**

Cross-sectional study of an age-stratified random sample of a population register.

**Setting:**

The city of Izhevsk, The Russian Federation, 2008–09.

**Participants:**

A total of 1031 men aged 25–60 years (68% response rate).

**Measurements:**

Self-reported health was evaluated with the SF12 physical (PCS) and mental (MCS) component summaries. Measures of hazardous drinking (based on frequency of adverse effects of alcohol intake including hangover, excessive drunkenness and extended episodes of intoxication lasting 2 or more days) were used in addition to frequency of alcohol consumption and total volume of beverage ethanol per year. Information on smoking and socio-demographic factors were obtained.

**Findings:**

Compared with abstainers, those drinking 10–19 litres of beverage ethanol per year had a PCS score 2.66 [95% confidence interval (CI) = 0.76; 4.56] higher. Hazardous beverage drinking was associated with a lower PCS score [mean diff: −2.95 (95% CI = −5.28; −0.62)] and even more strongly with a lower MCS score [mean diff: −4.29 (95% CI = −6.87; −1.70)] compared to non-hazardous drinkers, with frequent non-beverage alcohol drinking being associated with a particularly low MCS score [−7.23 (95% CI = −11.16; −3.29)]. Adjustment for smoking and socio-demographic factors attenuated these associations slightly, but the same patterns persisted. Adjustment for employment status attenuated the associations with PCS considerably.

**Conclusion:**

Among working-aged male adults in Russia, hazardous patterns of alcohol drinking are associated with poorer self-reported physical health, and even more strongly with poorer self-reported mental health. Physical health appears to be lower in those reporting complete abstinence from alcohol compared with those drinking 10–19 litres per year.

## INTRODUCTION

Following the collapse of the Soviet Union in 1991, Russia experienced huge fluctuations in life expectancy. These were driven largely by parallel fluctuations in alcohol-induced mortality that particularly affected working-aged men [1–6]. Although it has increased since 2005, Russia still has an exceptionally low life expectancy compared to the rest of Europe [7]. In 2010 life expectancy at birth in Russia was 63 years for males and 75 for females, compared to 79 and 83 years, respectively, for the United Kingdom.

Russians have a particularly hazardous pattern of alcohol consumption, characterized by a high prevalence of binge drinking and a high proportion of total consumption from spirits [8]. There is also widespread consumption of manufactured non-beverage alcohols sold as aftershaves, medicinal tinctures and as solvents [9–11], the consumption of which has been associated with particularly high mortality [2].

As well as having very high mortality at working ages, out of 18 European countries Russia has been found to have the highest prevalence of self-reported ill-health [12]. However, while the contribution of alcohol to high mortality has been the focus of much research [5], only a small number of studies have investigated the association between alcohol and self-reported health in Russia [13–16]. These studies had a number of weaknesses: most treated self-reported health as a binary variable, and distinguished simply between drinkers and non-drinkers. None looked at the association with volume of ethanol consumed or employed alternative measures that captured hazardous drinking, as has been advocated in the Russian context [17]. Regardless of country, in fact, there is a relative paucity of research on the impact of alcohol on self-reported health and quality of life. This is being seen increasingly as problematic both in terms of understanding the broader impact of alcohol on wellbeing as well as in intervention research, where it is argued that quality of life *per se* should be a key outcome criterion [18,19].

Of the studies that have looked at self-reported health or quality of life in relation to alcohol, very few have distinguished between the physical and mental health domains. The assumption implicit in considering a single combined measure of self-reported health is that alcohol has the same impact on both. However, this crucial assumption is largely untested. If there are indeed differential effects, the focus of interventions and services aimed at improving or ameliorating poor quality of life related to alcohol would need to reflect this. Poor self-reported mental health could, in principle, coexist with average or better physical health or vice versa.

In this paper we report on the association of patterns of drinking behaviour and self-reported health in Russia—a country with one of the most hazardous drinking patterns in the world. The study adds to the sparse literature in this area and overcomes some of the limitations of previous work, not least by treating the physical and mental component summary from the short form 12-item questionnaire (SF12) instrument as separate outcomes.

## METHODS

### Study design

The data analysed were from the main phase of the Izhevsk Family Study 2 (IFS2). Conducted in 2008–09, this was a cross-sectional survey of 1515 working-aged men (aged 25–60 years), 1031 of whom had a health check examination. Izhevsk is the capital city of the Udmurt republic, part of the Russian Federation, located 1300 km south east of Moscow. With a population of 611 000 in 2009, it has a typical demographic profile for a medium-sized Russian city. Life expectancy at birth is very similar to the national average, in 2009 being 63 years for males and 76 years for females. Participants were recruited originally (2003–05) as an age-stratified random sample (*n* = 2041) of a 2002 population register of adult city residents, the majority of whom were used as live controls in an earlier case–control study of premature mortality [2].

The IFS2 fieldwork had two parts. Initially a team of sociologists attempted to locate the current address of each man, and then sought to interview them (interview 2008/09). Once a man had been interviewed they were offered a physical examination (health check) that was carried out typically 3–4 weeks later (interquartile range: 20–42 days), if the man had provided signed consent.

### Socio-demographic and alcohol variables

At the interview information was obtained on socio-demographic characteristics such as age, educational level, employment status, nationality and whether or not the man's household had access to a car and/or central heating (household amenities). Information on smoking status was collected, as was information on indicators of health status, such as breathlessness on walking and recent weight loss.

Information on alcohol consumption was collected with a reference period of the previous year. Questions on frequency of intake of beer, wine, spirits and other alcoholic beverages were asked, together with the usual amount of beer, wine or spirits consumed on a typical occasion. The total volume of ethanol from beer, wine and spirits consumed in a year was estimated using the standard quantity–frequency approach [20]. The usual amount was obtained from the questionnaire using quantity units used commonly in Russia (bottles of beer, grams of wine and spirits). Beer was estimated to have an ethanol content of 4.5%, wine 12% and spirits 43% ethanol.

Information on frequency of hangover, excessive drunkenness or going to sleep at night clothed because of being drunk was also collected, as was frequency of *zapoi.* This latter term denotes a well-recognized phenomenon in Russia, where a person has a period of continuous drunkenness of 2 or more days, when they are withdrawn from normal social life. Based on these variables, an individual was classified as being a hazardous drinker if they had one or more of the following characteristics: twice-weekly or more occurrence of excessive drunkenness, hangover, going to sleep at night clothed because of being drunk or having one or more episodes of *zapoi* in the past year. This measure has been shown to be highly predictive of mortality in Russia [2,21] and has the advantage of being defined in terms of the frequency of readily observed behaviours that are going to be less subject to misclassification than specification of ‘usual’ volume of beer wines and spirits [22].

Information was also collected on frequency of consumption of non-beverage alcohols. As non-beverage alcohol is of widely varying concentration and comes in many different-sized bottles, the volume of ethanol from this source could not be estimated, and so the total volume of ethanol would be underestimated for non-beverage alcohol drinkers. Hence, these subjects were excluded for the analyses involving total annual volume of ethanol. However, information on whether the man had drunk non-beverage alcohol in the previous year was combined with whether or not they were a hazardous drinker (as defined above) to produce a four-level ‘class of drinker’ variable: abstainer, non-hazardous beverage drinker, hazardous beverage drinker and non-beverage alcohol drinker.

### Self-reported health variables

At the health check, participants were requested to self-complete a Russian version of the SF12 instrument. The SF12 was developed in 1994 in English as a shorter alternative to the SF36, and was translated subsequently into Russian and other languages as part of the International Quality of Life Project Assessment [23]. The SF12 is comprised of eight subscales: physical functioning, role (physical), bodily pain, general health, vitality, social functioning, role (emotional) and mental health. These were summarized into two scales: a physical component score (PCS) and a mental component score (MCS), in accordance with the guidelines for the SF12 instrument [24]. Both scores ranged between 0 and 100, with a higher score indicating better health. These SF12-based summaries have been shown to reproduce accurately both the PCS and the MCS derived from the full SF36 [25].

### Statistical methods

Mean differences [with 95% confidence intervals (CI)] for the association between measures of alcohol consumption and both the PCS and MCS were estimated using linear regression. Four models were constructed adjusting for potential confounders, all of which were treated as categorical variables: model 1, adjusting for age grouped into 5-year categories except the youngest age category, which was a 10-year age-band; model 2 for age and smoking; model 3 for age, smoking, education, household car/central heating and nationality; and model 4 is adjusted further for employment status. *P*-values for both heterogeneity and linear trend were obtained using the partial *F*-test. Men with missing values in any variable were excluded from the analyses. A priori interactions between age and all measures of alcohol were investigated with respect to both the PCS and the MCS. The linear regression assumption of normal distribution of residuals was checked by inspection of the standardized residuals in qq-plots and in histograms. The assumption of equal variances of the residuals was checked by visual inspection. All analyses were conducted in Stata version 11 [26].

### Ethical approval

Ethical approval for the Izhevsk Family Study was granted by the Izhevsk State Medical Academy Ethics Committee on 23 October 2007 and the LSHTM Ethics Committee on 16 January 2008.

## RESULTS

A total of 1515 men completed an interview in 2008/9. Of these, 1052 (69%) attended a health check, among whom 1031 (68%) provided sufficient information to derive an SF12 score. Among those with an SF12 score, mean age at interview was 48 years; the majority were drinkers (87%), among whom the mean intake of ethanol was 9.5 litres/year per person [standard deviation (SD) 11.4]. Abstainers included both life-long abstainers (1% of the total population) and former drinkers (12.1% of the total population).

The percentages of those with an SF12 score by characteristics determined at interview 2008/09, along with the mean PCS and MCS scores by alcohol consumption and co-variables, are shown in Table 1. The only variable that showed strong evidence of being related to having an SF12 score was the measure of household access to cars and central heating. Importantly, there was no systematic or substantial variation in percentage with an SF12 score according to alcohol consumption.

**Table 1:** The response to the short form (SF)12 questionnaire and the mean of the SF12 physical and mental component summary distributed on characteristics of a sample of Russian men aged 25–60 years

	*Number of men with SF12/total subjects at interview 2008/09 (%)*	*Mean physical component score (SD)*	*Mean mental component score (SD)*
Frequency of alcohol consumption (any kind)			
Daily or nearly every day	85/131 (64.9)	47.7 (6.88)	47.2 (9.45)
1–4 times per week	482/719 (67.0)	48.0 (8.22)	48.5 (8.62)
1–3 times a month or less	330/461 (71.6)	47.0 (8.80)	48.6 (9.12)
Abstainer	133/202 (65.8)	45.4 (8.86)	48.2 (9.89)
Missing	1/2	1	1
*P-*value heterogeneity	0.26^a^	0.016^b^	0.59^b^
Total volume beverage ethanol (litres per year)			
Abstainer	133/ 202 (65.8)	45.4 (8.86)	48.2 (9.89)
<2 litres	190/273 (69.9)	46.6 (8.99)	49.1 (9.54)
2–4 litres	207/303 (68.3)	47.7 (8.29)	48.6 (8.24)
5–9 litres	199/289 (68.9)	48.1 (8.04)	48.5 (8.83)
10–19 litres	170/246 (69.1)	48.3 (8.06)	48.5 (8.47)
20+ litres	119/181 (65.8)	47.2 (8.15)	46.6 (9.54)
Missing	13/21	13	13
*P-*value heterogeneity	0.92^a^	0.029^b^	0.29^b^
Class of drinker			
Abstainer	133/202 (65.8)	45.4 (8.86)	48.2 (9.89)
Non-hazardous beverage drinkers	783/1145 (68.4)	47.9 (8.14)	49.0 (8.67)
Hazardous beverage drinkers	49/67 (73.1)	45.7 (9.43)	44.6 (9.70)
Non-beverage alcohol drinkers	50/76 (65.8)	45.3 (8.60)	44.3 (9.18)
Missing	16/25	16	16
*P-*value heterogeneity	0.69^a^	0.0014^b^	<0.001^b^
Frequency of non-beverage alcohol consumption			
Beverage drinkers	845/1232 (68.6)^c^	47.7 (8.30)^d^	48.6 (8.81)^d^
Monthly or less	32/48 (66.7)	46.2 (8.24)	46.8 (8.04)
Daily to weekly	20/32 (62.5)	43.5 (8.79)	41.8 (10.75)
Missing	1/1	1	1
*P-*value heterogeneity	0.74^a^	0.054^b^	0.002^b^
Age (years)			
25–34	92/132 (69.7)	51.0 (7.27)	46.0 (10.56)
35–39	96/142 (67.6)	50.0 (7.58)	49.1 (7.62)
40–44	119/164 (72.6)	49.8 (7.19)	48.6 (8.04)
45–49	198/281 (70.5)	48.2 (7.79)	48.0 (8.83)
50–54	255/370 (68.9)	45.7 (8.82)	48.8 (9.27)
55–60	271/426 (63.6)	44.9 (8.59)	48.7 (9.18)
Missing	0/0	0	0
*P-*value heterogeneity	0.27^a^	<0.001^b^	0.13^b^
Smoking			
Never smoked	198/292 (67.8)	48.9 (8.67)	48.9 (8.75)
Ex-smoker	185/264 (70.1)	46.8 (8.54)	48.6 (8.67)
1–10/day	119/166 (71.7)	47.7 (9.94)	48.3 (9.33)
11–20/day	403/606 (66.5)	46.7 (8.04)	48.2 (9.28)
>20/day	125/186 (67.2)	47.3 (7.24)	48.0 (8.93)
Missing	1/1	1	1
*P-*value heterogeneity	0.69^a^	0.037^b^	0.86^b^
Education			
Incomplete secondary	46/71(64.8)	45.5 (8.97)	48.6 (10.78)
Secondary	754/1100 (68.6)	46.7 (8.54)	48.5 (9.08)
Higher	231/343 (67.4)	49.5 (7.53)	47.8 (8.47)
Missing	0/0	0	0
*P-*value heterogeneity	0.76^a^	<0.001^b^	0.59^b^
Household has car and/or central heating			
Neither	62/116 (53.5)	46.3 (9.51)	48.2 (10.96)
Only one	481/726 (66.3)	46.5 (8.56)	47.5 (9.20)
Both	488/673 (72.5)	48.2 (8.05)	49.2 (8.51)
Missing	0/0	0	0
*P-*value heterogeneity	<0.001^a^	0.003^b^	0.012^b^
Nationality			
Russian	667/977 (68.3)	47.5 (8.46)	48.4 (8.94)
Udmurt	232/333 (69.7)	46.7 (7.91)	48.1 (9.44)
Tartar	95/152 (62.5)	46.9 (9.37)	48.4 (9.19)
Other	37/52 (71.2)	47.7 (8.37)	50.5 (7.49)
Missing	0/1	0	0
*P-*value heterogeneity	0.42^a^	0.58^b^	0.51^b^
In employment			
Yes	863/1254 (68.8)	48.2 (7.57)	48.9 (8.51)
No	168/261 (64.4)	42.8 (10.83)	45.6 (10.94)
Missing	0/0	0	0
*P-*value heterogeneity	0.16^a^	<0.001^b^	<0.001^b^
Climb up a flight of stairs without breathlessness in recent months			
Yes, easily	934/1366 (68.4)	48.6 (7.25)	48.9 (8.66)
Yes, with some difficulty	78/118 (66.1)	36.5 (9.01)	44.9 (9.28)
No, too difficult	18/29 (62.1)	30.2 (9.78)	36.7 (14.41)
Missing	1/2	1	1
*P-*value heterogeneity	0.69^a^	<0.001^b^	<0.001^b^
Weight loss during the past year			
No	903/1320 (68.4)	47.6 (8.08)	48.8 (8.81)
Yes	121/182 (66.5)	45.0 (10.28)	45.8 (10.07)
Missing	7/13	7	7
*P-*value heterogeneity	0.60^a^	0.001^b^	<0.001^b^
Total	1031/1515 (68.1)	47.31 (8.42)	48.38 (9.03)

SD = standard deviation.

a *P*-value from the χ^2^ test;

b *P*-value from one-way analysis of variance (ANOVA) test;

c 202 abstainers excluded;

d 133 abstainers excluded.

Table 2 presents age-adjusted differences in PCS and MCS by categories of the non-alcohol variables from Table 1. The face validity of PCS was suggested by the strong evidence of an association with breathlessness and recent weight loss in the expected direction. Although MCS showed a similar association with these aspects of physical health, we were unable to demonstrate directly its face validity *per se*, as no equivalent mental health variables were available in the study. PCS was related to smoking, education, household amenities and employment status, but MCS was related only to the last two of these.

**Table 2:** The association between smoking, socio-demographic factors and health questions, and the physical and mental component summary adjusted for age in a sample of Russian men aged 25–60 years

	*n*	*PCS*	*MCS*
		*Mean diff.*^a^*(95% CI)*^b^	*Mean diff.*^a^*(95% CI)*^b^
Smoking	1030		
Never smoked	198	0 [Ref]	0 [Ref]
Ex-smoker	185	−1.60 (−3.23;0.03)	−0.45 (−2.26;1.37)
1–10/day	119	1.20 (−3.06;0.65)	−0.74 (−2.80;1.32)
11–20/day	403	−2.09 (−3.47;−0.70)	−0.79 (−2.33;0.74)
>20/day	125	−2.09 (−3.91;−0.26)	−0.90 (−2.92;1.13)
*P-*value for heterogeneity^c^		0.047	0.87
*P-*value for trend^c^		0.007	0.28
Education	1031		
Incomplete secondary	46	−0.21 (−2.64;2.22)	0.06 (−2.64;2.77)
Secondary	754	0 [Ref]	0 [Ref]
Higher	231	2.32 (1.12;3.52)	−0.56 (−1.90;0.77)
*P-*value for heterogeneity^c^		<0.001	0.70
*P-*value for trend^c^		<0.001	0.42
Household has car and/or central heating	1031		
Neither	62	−0.43 (−2.58;1.71)	0.81 (−1.57;3.19)
Only one	481	0 [Ref]	0 [Ref]
Both	488	1.75 (0.73;2.77)	1.71 (0.58;2.84)
*P-*value for heterogeneity^c^		0.002	0.013
*P-*value for trend^c^		<0.001	0.003
Nationality^e^	1031		
Russian	667	0 [Ref]	0 [Ref]
Udmurt	232	−0.62 (−1.84;0.61)	−0.31 (−1.66;1.04)
Tartar	95	−0.54 (−2.30;1.22)	0.11 (−1.84;2.06)
Other	37	0.28 (−2.42;2.99)	1.98 (−1.01;4.97)
*P-*value for heterogeneity^c^		0.73	0.56
In employment	1031		
Yes	863	0 [Ref]	0 [Ref]
No	168	−4.77 (−6.11;−3.44)	−3.47 (−4.97; −1.98)
*P-*value heterogeneity^c^		<0.001	<0.001
Climb up a flight of stairs without breathlessness in recent months	1030		
Yes, easily	934	0 [Ref]	0 [Ref]
Yes, with some difficulty	78	−11.24 (−12.94;−9.54)	−4.26 (−6.32;−2.21)
No, too difficult	18	−17.31 (−20.72;−13.90)	−12.63 (−16.75;−8.50)
*P-*value for heterogeneity^c^		<0.001	<0.001
*P-*value for trend^c^		<0.001	<0.001
Weight loss during the past year	1024		
No	903	0 [Ref]	0 [Ref]
Yes	121	−2.66 (−4.20;−1.13)	−2.92 (−4.63;−1.22)
*P-*value for heterogeneity^c^,^d^		<0.001	<0.001

All models are adjusted for age using linear regression.

a Mean diff. = mean difference from reference group;

b 95% CI = 95% confidence interval for the mean difference;

c from the partial *F*-test;

d *P*-value for trend omitted because of binary variable;

e *P*-value for trend omitted because of non ordered categories. [Ref] = reference group. PCS = physical component score; MCS = mental component score.

The adjusted associations between level and pattern of alcohol consumption and the PCS and MCS scores are shown in Tables 3 and 4, respectively. Compared to regular drinkers, those who abstain or drink a few times a month or less had poorer physical health (Table 3). Parallel to this, in models 2 and 3 there was a significant trend of increasing PCS score as annual volume of ethanol increased, although there was some indication of a decline in score for men who drank 20+ versus those drinking 2–19 litres per year. However, hazardous alcohol drinkers had lower PCS scores than non-hazardous drinkers. Frequency of non-beverage alcohol consumption was not related to PCS, although this study has limited power to detect small or moderate effects due to the relatively small number of non-beverage drinkers. These patterns were attenuated only slightly after adjustment for smoking, education, nationality and household amenities. However, additional adjustment for employment status reduced the strength of associations of alcohol with PCS considerably (Table 3).

**Table 3:** The association between the physical component summary and alcohol consumption with and without adjustment for smoking and socio-demographic factors in a sample of Russian men aged 25–60 years

	n	*Model 1*	*Model 2*	*Model 3*	*Model 4*
		*Mean diff.*^a^*(95% CI)*^b^	*Mean diff.*^a^*(95% CI)*^b^	*Mean diff.*^a^*(95% CI)*^b^	*Mean diff.*^a^*(95% CI)*^b^
Frequency of alcohol consumption (any kind)	1028				
Daily or nearly every day	85	0 [Ref]	0 [Ref]	0 [Ref]	0 [Ref]
1–4 times per week	481	0.71 (−1.18; 2.60)	0.46 (−1.45; 2.37)	0.21(−1.70; 2.12)	0.30 (−1.58; 2.18)
1–3 times a month or less	330	0.17 (−1.79; 2.13)	−0.35 (−2.35; 1.66)	−0.65(−2.66; 1.37)	−0.45 (−2.43; 1.53)
Abstainer	132	−1.54 (−3.78; 0.70)	−1.80 (−4.06; 0.46)	−1.79 (−4.05; 0.48)	−1.49 (−3.71; 0.73)
*P-*value for heterogeneity^c^		0.048	0.042	0.077	0.13
*P-*value for trend^c^		0.038	0.015	0.018	0.037
Total volume beverage ethanol (litres per year)^d^	967				
Abstainer	132	0 [Ref]	0 [Ref]	0 [Ref]	0 [Ref]
<2 litres	186	1.57 (−0.26; 3.40)	1.18 (−0.66; 3.02)	0.80 (−1.05; 2.64)	0.71 (−1.10; 2.52)
2–4 litres	201	2.23 (0.43; 4.02)	2.01 (0.22; 3.81)	1.65 (−0.15; 3.46)	1.57 (−0.20; 3.33)
5–9 litres	191	2.35 (0.54; 4.17)	2.35 (0.54; 4.17)	2.06 (0.24; 3.87)	1.74 (−0.04; 3.52)
10–19 litres	156	2.66 (0.76; 4.56)	2.74 (0.84; 4.64)	2.50 (0.61; 4.40)	1.98 (0.12; 3.85)
20+ litres	101	1.30 (−0.83; 3.43)	1.50 (−0.64; 3.64)	1.22 (−0.93; 3.36)	0.99 (−1.12; 3.09)
*P-*value for heterogeneity^c^		0.073	0.063	0.11	0.26
*P-*value for trend^c^		0.06	0.018	0.025	0.079
Class of drinker^e^	1013				
Abstainer	132	−2.32 (−3.81; −0.83)	−2.23 (−3.72; −0.74)	−1.93 (−3.43; −0.44)	−1.66 (−3.14; −0.18)
Non-hazardous beverage drinkers	782	0 [Ref]	0 [Ref]	0 [Ref]	0 [Ref]
Hazardous beverage drinkers	49	−2.95 (−5.28; −0.62)	−2.65 (−5.00; −0.31)	−2.50 (−4.85; −0.15)	−1.87 (−4.19; 0.45)
Non-beverage alcohol drinkers	50	−2.02 (−4.32; 0.29)	−1.69 (−4.01; 0.64)	−1.05 (−3.40; 1.30)	−0.24 (−2.57; 2.08)
*P-*value for heterogeneity^c^		0.001	0.004	0.018	0.08
*P-*value for trend^c^ (not including abstainers)		0.011	0.032	0.14	0.47
Frequency of non-beverage alcohol consumption^f^	897				
Beverage drinkers	845	0 [Ref]	0 [Ref]	0 [Ref]	0 [Ref]
Monthly or less	32	−1.41 (−4.26; 1.43)	−1.27 (−4.10; 1.57)	−0.47 (−3.33; 2.38)	−0.43 (−3.24; 2.38)
Daily to weekly	20	−2.70 (−6.29; 0.88)	−2.39 (−5.98; 1.19)	−1.61 (−5.20; 1.98)	0.37 (−3.24; 3.97)
*P-*value for heterogeneity^c^		0.21	0.30	0.65	0.93
*P-*value for trend^c^		0.081	0.12	0.82	0.99

Model 1: adjusted for age (in 5-year categories); model 2: adjusted for age and smoking; model 3: adjusted for age, smoking, education, car and heating and nationality; model 4: adjusted for age, smoking, education, car and heating, nationality and employment. All models are adjusted using linear regression.

a Mean difference from reference group;

b 95% confidence interval for mean difference;

c from the partial *F*-test;

d 50 non-beverage alcohol consumers are excluded from this variable;

e *P*-value for interaction between class of drinker and age: *P* = 0.027;

f 132 abstainers are excluded from this variable. [Ref] = reference group; CI = confidence interval.

**Table 4:** The association between the mental component summary and alcohol consumption with and without adjustment for smoking and socio-economic factors in a sample of Russian men aged 25–60 years

	*n*	*Model 1*	*Model 2*	*Model 3*	*Model 4*
		*Mean diff.*^a^*(95% CI)*^b^	*Mean diff.*^a^*(95% CI)*^b^	*Mean diff.*^a^*(95% CI)*^b^	*Mean diff.*^a^*(95% CI)*^b^
Frequency of alcohol consumption (any kind)	1028				
Daily or nearly every day	85	0 [Ref]	0 [Ref]	0 [Ref]	0 [Ref]
1–4 times per week	481	1.26 (−0.83; 3.35)	1.18 (−0.94; 3.31)	0.83 (−1.30; 2.96)	0.90 (−1.22; 3.01)
1–3 times a month or less	330	1.19 (−0.98; 3.36)	1.02 (−1.21; 3.25)	0.65 (−1.59; 2.90)	0.81 (−1.42; 3.03)
Abstainer	132	0.89 (−1.59; 3.37)	0.79 (−1.73; 3.30)	0.44 (−2.09; 2.96)	0.66 (−1.84; 3.16)
*P-*value for heterogeneity^c^		0.68	0.74	0.88	0.87
*P-*value for trend^c^		0.76	0.88	0.97	0.84
Total volume beverage ethanol (litres per year)^d^	967				
Abstainer	132	0 [Ref]	0 [Ref]	0 [Ref]	
<2 litres	186	0.92 (−1.09; 2.94)	0.86 (−1.18; 2.90)	0.87 (−1.18; 2.92)	0.81 (−1.22; 2.84)
2–4 litres	201	0.41 (−1.57; 2.38)	0.39 (−1.60; 2.38)	0.48 (−1.52; 2.48)	0.42 (−1.57; 2.40)
5–9 litres	191	0.47 (−1.53; 2.46)	0.48 (−1.52; 2.50)	0.51 (−1.50; 2.52)	0.28 (−1.72; 2.27)
10–19 litres	156	0.74 (−1.35; 2.83)	0.77 (−1.33; 2.87)	0.70 (−1.41; 2.80)	0.32 (−1.78; 2.41)
20+ litres	101	−0.53 (−2.87; 1.82)	−0.48 (−2.85; 1.89)	−0.17 (−2.56; 2.21)	−0.34 (−2.71; 2.02)
*P-*value for heterogeneity^c^		0.82	0.86	0.93	0.94
*P-*value for trend^c^		0.72	0.82	0.91	0.66
Class of drinker	1013				
Abstainer	132	−0.82 (−2.47; 0.83)	−0.81 (−2.47; 0.85)	−0.85 (−2.52; 0.82)	−0.66 (−2.32; 1.01)
Non-hazardous beverage drinkers	782	0 [Ref]	0 [Ref]	0 [Ref]	0 [Ref]
Hazardous beverage drinkers	49	−4.29 (−6.87; −1.70)	−4.22 (−6.83; −1.62)	−3.97 (−6.59; −1.35)	−3.53 (−6.14; −0.92)
Non-beverage alcohols drinkers	50	−4.91 (−7.47; −2.36)	−4.83 (−7.41; −2.24)	−4.58 (−7.20; −1.97)	−4.03 (−6.65; −1.41)
*P-*value for heterogeneity^c^		<0.001	<0.001	<0.001	0.002
*P-*value for trend^c^ (not including abstainers)		<0.001	<0.001	<0.001	<0.001
Frequency of non-beverage alcohol consumption^e^	897				
Beverage drinkers	845	0 [Ref]	0 [Ref]	0 [Ref]	
Monthly or less	32	−2.08 (−5.20; 1.04)	−2.00 (−5.14; 1.14)	−1.86 (−5.04; 1.31)	−1.83 (−4.99; 1.32)
Daily to weekly	20	−7.23 (−11.16; −3.29)	−7.15 (−11.17; −3.13)	−6.89 (−10.87; −2.90)	−5.49 (−9.53; −1.45)
*P-*value for heterogeneity^c^		0.001	0.001	0.002	0.018
*P-*value for trend^c^		<0.001	<0.001	0.001	0.005

Model 1: adjusted for age (in 5-year categories); model 2: adjusted for age and smoking; model 3: adjusted for age, smoking, education, car and central heating and nationality; model 4: adjusted for age, smoking, education, car and heating, nationality and employment. All models are adjusted using linear regression.

a Mean difference from reference group;

b 95% confidence interval for mean difference;

c from the partial *F*-test;

d 50 non-beverage alcohol consumers are excluded from this variable;

e 132 abstainers are excluded from this variable. [Ref] = reference group; CI = confidence interval.

MCS scores were not related to either frequency of alcohol consumption or total volume of ethanol consumed (Table 4). However, hazardous drinkers and those who drink non-beverage alcohols had lower MCS scores than non-hazardous drinkers or abstainers. There was strong evidence that MCS scores decline with frequency of drinking of non-beverage alcohols. Adjustment for smoking and socio-demographic variables attenuated the associations with MCS to some degree, but they remained strong and highly significant.

No age-interaction was detected except for the association of class of drinker with PCS (*P* = 0.03). It was found that hazardous beverage drinking reduced the PCS score only among men aged less than 45 years. In this younger age group, hazardous beverage drinkers had a PCS score that was −6.82 (95% CI = −10.42, –3.23) relative to non-hazardous beverage drinkers. However, among older men there was no evidence of an association of PCS with class of drinker.

As the residuals of models with the PCS were moderately skewed to the left, we carried out a sensitivity analysis with a square-transformed PCS. As the conclusions were essentially unchanged, and because linear regression is fairly robust to departure from normality, the PCS were kept on a normal linear scale. The residuals for models with the MCS were approximately normally distributed and there were no patterns in the variance. The variance assumptions for key exposures were overall met by visual inspections.

## DISCUSSION

In this first study, to examine in detail the association of drinking with self-reported health in Russia we found striking patterns that differed, both according to different measures of drinking behaviour and outcome (self-reported physical versus mental health). Physical health scores were highest for those who reported drinking several times a week and lowest for abstainers—almost all of whom were former drinkers. Most strikingly, the physical score increased almost monotonically from abstainers to those drinking 10–19 litres per year, then declined slightly for the heaviest drinkers. In contrast, mental health scores showed no relationship with either frequency or annual volume consumed. The culturally specific measure of hazardous drinking, however, was associated with poorer physical health and even more strongly with poorer mental health in the anticipated direction, with frequent non-beverage drinking being associated with particularly poor mental health. None of these effects appear to be explained by confounding by smoking, education, nationality or household amenities.

Additional adjustment for employment status attenuated the associations more than the other socio-demographic variables, although the mental health associations with hazardous and non-beverage alcohol drinking remained strong even in this final model. Care needs to be taken when interpreting the attenuation of the associations. As argued elsewhere [2], because heavy drinking is likely, in itself, to result in an increased risk of falling out of employment, knowledge about employment status might carry information about drinking history and behaviours above and beyond that contained in the variables that attempt to measure alcohol drinking patterns and behaviours directly. To this extent, it could be that including employment in the regression model results in over-adjustment.

To our knowledge, this paper is the first to investigate the association of alcohol drinking with the SF12 physical and mental component scores in Russia. One previous study in Russia used the more extensive SF36 instrument [15], and found that ‘poor physical functioning’ declined as frequency of alcohol consumption increased. This is consistent with our finding. However, the top frequency category in this study was defined as drinking more than once per week, and so any adverse effects of very frequent drinking (daily or almost daily) could not be identified.

The other studies of alcohol in relation to self-reported health in Russia have focused on a binary variable of ‘poor health’ derived from a five-point scale (e.g. very good, good, average, poor, very poor) [13–16]. Three of these found evidence of a U-or J-shaped association of frequency of drinking with risk of poor self-reported health [14–16]. Nicholson *et al*. investigated drinking more than 0.5 litres of vodka in one evening, a measure of hazardous drinking, and found an elevated odds ratio for poor self-reported health, although the confidence intervals for this finding were overlapping [14]. It should be noted that this study did not investigate the age-specific effects of hazardous alcohol drinking. In relation to this, we found that hazardous drinking and self-reported physical health was very strongly associated among younger men. Hazardous drinking behaviour is less common among younger men, and to this extent could be seen to be particularly extreme and likely to be damaging. No studies in a Russian context have investigated self-reported mental health in relation to alcohol consumption.

How do our findings in this very heavy drinking population fit within the broader, although sparse literature? In a large UK-based study [27], improvements in both SF36 mental and physical component scores were seen with increasing volume of consumption. However, volume was analysed on a limited, three-point scale, with the top category being greater or equal to 3.6 litres/year—far below the level at which we observed a downturn in PCS score.

The most comprehensive analysis of SF36 physical and mental component scores in relation to different measures of drinking was conducted in the Western New York Health Study [28]. This study showed that drinking to intoxication once or more in the past 30 days was associated with poorer physical but not mental health. In comparison to that, our finding of a strong association of mental health with hazardous drinking is somewhat surprising, and it should be borne in mind that the particular measure we used is culturally specific and has a higher threshold for the determination of hazardous drinking. Also, the absence of a strong effect on mental health in the New York study might simply be because hazardous drinking was less prevalent than in Izhevsk. Evidence from studies conducted in the United States and Holland also suggest that SF36 mental health measures are associated with alcohol dependence [29,30]. This evidence provides support for the expectation that associations with self-reported health will be correlated negatively more strongly among those with more hazardous and harmful drinking than at lower levels.

There are a number of explanations for the association of hazardous drinking behaviour with self-reported mental health. Alcohol might be used as self-medication in order to relieve distress from mental disorders such as anxiety and depression. Conversely, alcohol might provoke mental distress in sensitive individuals [31]. In our study, and most others looking at this issue, it was not possible to distinguish these two explanations, due to the limitations of the cross-sectional design. The issue of reverse causality in relation to depression and alcohol consumption has been investigated extensively without any clear conclusion [32–35]. Social disadvantage could also be a determinant of both alcohol problems [36] and mental disorders [37]. However, in our study and a number of others the associations were detected independently of socio-demographic factors. Finally, it is possible that reporting behaviour accounts for at least some of these findings.

With the exception of the New York study, the findings from the present study are thus consistent with the few previous studies in not showing deterioration in self-reported physical health as alcohol consumption increased. This is prima facie surprising and, interestingly, our mental health findings are more in line with what would have been expected, with poorer mental health reported as alcohol consumption increased, particularly at the higher end.

Our study has a number of strengths and weaknesses. As was the case in all the other studies cited above that reported response rates, there is a concern that both heavy drinkers and those with poor health were excluded differentially from the study. However, we found that non-response from the interview 2008/09 to the health check examination was not related to alcohol consumption or health indicators. Nevertheless, non-response to the interview 2008/09 could introduce bias.

A strength of the study is that we used a variety of different measures of alcohol consumption. This enabled us to detect specific associations of poor mental health with markers of hazardous drinking that were not evident using frequency or volume of ethanol variables, thus demonstrating the contingency of these associations on the particular measures used or aspects of drinking being studied.

The study population was sampled among men from one Russian city and was restricted in the initial sampling (2003–05) to men living in a household with others. Hence, caution must be taken when generalizing the results of our study to Russia as a whole. Nevertheless, the results of this study suggest that the relatively common pattern of hazardous drinking found among Russian men is associated with an increased burden of self-reported mental ill-health. Undertaking longitudinal studies is a priority to establish definitively the direction of causality underlying these associations.

The implications of these data warrant careful consideration. Measures such as SF12 and SF36 are used as outcomes in both brief intervention and treatment evaluation studies. However, interventions successfully altering drinking behaviour might not show anticipated improvements in self-reported physical health; indeed, it seems possible that adverse effects could be reported. Hence, future studies of self-reported health in relation to alcohol need to use a more nuanced approach to characterizing consumption patterns as well as to differentiate between self-reported mental and physical ill-health. This is important to pursue, as decisions about health-care resource use are based increasingly on generic health outcome measures.
